# Inhibition of Y1 Receptor Promotes Osteogenesis in Bone Marrow Stromal Cells *via* cAMP/PKA/CREB Pathway

**DOI:** 10.3389/fendo.2020.583105

**Published:** 2020-11-10

**Authors:** Wei Yu, Fan-Cheng Chen, Wen-Ning Xu, Sheng-Long Ding, Peng-Bo Chen, Lei Yang, Sheng-Dan Jiang, Xiao-Yun Pan

**Affiliations:** ^1^ Department of Orthopaedic Surgery, Second Affiliated Hospital and Yuying Children's Hospital of Wenzhou Medical University, Wenzhou, China; ^2^ Department of Clinic of Spine Center, Xinhua Hospital, Shanghai Jiaotong University School of Medicine, Shanghai, China; ^3^ Shanghai Medical College, Fudan University, Shanghai, China; ^4^ Department of Orthopaedic, Qingpu Branch of Zhongshan Hospital, Fudan University, Shanghai, China

**Keywords:** neuropeptide Y1 receptor, RUNX2, osteogenesis, bone marrow stromal cells (BMSCs), protein kinase A

## Abstract

Inhibition of neuropeptide Y1 receptor stimulates osteogenesis *in vitro* and *in vivo*. However, the underlying mechanisms involved in these effects remain poorly understood. Here we identify the effects of Y1 receptor deficiency on osteogenic differentiation in human bone marrow stromal cells (BMSCs) by using genetic and pharmacological regulation, and to explore the pathways mediating these effects. In BMSCs, inhibition of Y1 receptor stimulates osteogenesis and upregulates the expression levels of the master transcriptional factor RUNX2. Mechanistically, Y1 receptor deficiency increases the levels of intracellular cAMP, which *via* protein kinase A (PKA) mediated pathways results in activation of phospho-CREB (p-CREB). We find RUNX2 activation induced by Y1 receptor deficiency is reversed by H-89, a PKA inhibitor. These results indicate Y1 receptor deficiency activates PKA-mediated phosphorylation of CREB, leading to activation of RUNX2 and enhances osteogenic differentiation in BMSCs. In conclusion, these data indicate that Y1 receptor deficiency promotes osteogenic differentiation by RUNX2 stimulation through cAMP/PKA/CREB pathway.

## Introduction

Neuropeptide Y receptors belong to the G protein-coupled receptor (GPCR) family and are widely expressed in the central nervous system and peripheral tissues (1, 2). Among the five NPY receptors (Y1, Y2, Y4, Y5, and Y6 receptors), Y1 is the first characterized receptor which plays important roles in cancer (1), energy homeostasis (3), and bone metabolism (4). *In vivo*, Y1 receptor ablation enhances osteoblast differentiation and bone formation, thus results in a high bone mass phenotype of mice (5). Pharmacological blockade of Y1 receptors by antagonists also improves bone mass in mice (6). *In vitro*, bone marrow-derived mesenchymal stem cells (BMSCs) isolated from Y1 receptor deletion mice show greater potentials to differentiated into mature osteoblast under the conditioned medium (7). Furthermore, We found that Y1 receptor knockdown rescued osteogenesis inhibition induced by glucocorticoid in preosteoblastic MC3T3-E1 cells (8). These findings together suggest Y1 receptor deficiency promotes osteogenesis. However, the underlying mechanisms involved in these effects remain elusive.

Human bone marrow stromal cells (hBMSCs) have the potential to differentiate into the osteogenic lineage given the appropriate conditions (9). This process is regulated by numerous osteogenesis factors including RUNX2, Osterix, Wnt-β-catenin, parathyroid hormone (PTH), and bone morphogenetic protein (BMP) (10). As the master transcriptional factor of osteogenesis, RUNX2 regulates osteogenic differentiation and is essential for normal skeletal formation as loss of RUNX2 in mice results in impaired postnatal skeletogenesis (11). RUNX2 regulates osteogenic differentiation by induction of specific osteoblast differentiation markers, namely, Osterix (12), ALP (alkaline phosphatase), OPN (osteopontin), BSP (bone sialoprotein), and OCN (osteocalcin) (13, 14).

Meanwhile, studies have showed that protein kinase A (PKA) signaling plays a critical role in osteogenic differentiation of BMSCs (15, 16). PKA is preferentially activated by increased level of cyclic adenosine monophosphate (cAMP), which then translocates into the nucleus and activates the cAMP response element-binding protein (CREB) at Ser-133. Activation of phospho-PKA (p-PKA) and phospho-CREB (p-CREB) contributes to osteogenic differentiation of BMSCs (15). The pro-osteogenic effects of cAMP/PKA/CREB signaling in BMSCs are associated with the induction of bone-related cytokines such as BMP2, TGF-β, IGF-1 (15), osteogenic markers including RUNX2 (17), and Osterix (18).

Interestingly, the association between NPY receptors and cAMP/PKA/CREB signaling has been widely reported. Activated by the ligands (neuropeptide Y, peptide YY, pancreatic polypeptide), Y receptors preferentially couple to G_i/o_ G-proteins, and thus inhibit adenylyl cyclase and lead to reduced cAMP level in islet, cancer, and fat tissues (1, 19, 20). Whereas Y1 receptor deficiency increases cAMP levels, which *via* PKA/CREB pathway results in improved outcome of islet transplantation (21). In osteoblastic cells, NPY is reported to reduce cAMP production and inhibit osteoblast differentiation (22, 23). Particularly, Y1 receptors are highly expressed in BMSCs. However, the interaction between cAMP/PKA/CREB signaling and Y1 receptor in the regulation of osteogenic differentiation in BMSCs remains unknown. Here we test whether the pro-osteogenic effects of Y1 receptor deficiency are associated with cAMP/PKA/CREB signaling.

In the present study, we hypothesize that inhibition of Y1 receptor signaling might increase RUNX2 levels and stimulate osteogenic differentiation due to the activation of cAMP/PKA pathway in human BMSCs.

## Materials and Methods

### Isolation and Culture of hBMSCs

Bone marrow aspirates (5 ml) were isolated from the posterior iliac crest from five healthy volunteers (3 males and 2 female; aged 20–40 years). The volunteers had similar lifestyles (low-moderate level of physical activity; no smoking habit), and all of them denied the history of long-term intake of calcium, vitamin D or other potentially nutrients influencing bone metabolism. For female subjects, the biopsy was obtained at the same stage of the period. Informed consent was obtained from all volunteers and the study was approved by the Institutional Review Board (IRB) of the Second Affiliated Hospital, Wenzhou Medical University (IRB No. 2018-14). hBMSCs were isolated as we previously described (24). Cells from each donor were cultured independently, and experiments were repeated at least 3 times. hBMSCs were cultured in Dulbecco’s modified Eagle media (DMEM, Gibco) supplemented with 10% fetal bovine serum (FBS, Gibco), 1% penicillin and streptomycin (Gibco) at 37°C in a 5% CO2 atmosphere. Cells grown to 80% to 90% confluency were subcultured by using 0.25% trypsin-EDTA (Gibco), and the third passage of cells was used in the following experiments. To determine the effects of Y1 receptor deficiency on osteogenic differentiation, hBMSCs were cultured in osteogenic media (growth culture media supplemented with 10^−8^ M dexamethasone (Sigma), 50 μg/ml ascorbic acid (Gibco) and 5 mM β-glycerol phosphate (Sigma) for 5 to 10 days. The media were refreshed twice a week during osteogenic differentiation.

### Cell Proliferation Assay

Cell proliferation rates were evaluated by using a cell counting kit (CCK-8, Dojindo), according to the manufacturer’s instructions. Briefly, cells were seeded in 96-well plates and each well was treated with 10 μl CCK-8 reagent for 4 h. Absorbance at 570 nm was read on a microplate reader (Bio-Rad). Cell proliferation rates were expressed as fold changes relative to the con groups.

### Alkaline Phosphatase/Alizarin Red S Staining and Assay

Alkaline phosphatase (ALP) staining was performed at day 5 in BMSCs during osteogenic differentiation. Cells rinsed with phosphate-buffered saline (PBS) were fixed with 4% paraformaldehyde, and then ALP staining was performed using an ALP substrate mixture (ALP staining kit, Sigma) for 30 min in darkness. ALP activity was colorimetrically determined at 405 nm using a Sigma kit with p-nitrophenyl phosphate (pNPP) as the substrate. The protein contents were measured using a protein assay kit (Bio-Rad) based on the bicinchoninic acid (BCA) method, and the overall enzyme activity was presented as units/mg protein.

Alizarin red S (ARS) staining was performed at day 10 during osteogenic differentiation. Cells were fixed in ice-cold 70% ethanol and then stained with 3% alizarin red S solution (Sigma) for 30 min. The bound dye was then colorimetrically detected at 595 nm after de-staining with 10% cetylpyridinium chloride monohydrate (Sigma) for 20 min. The quantification of ARS staining was conducted by drawing an Alizarin Red standard curve.

### Real-time Polymerase Chain Reaction (PCR) Assay

Real-time PCR was performed as previously reported (8), to detect the expression of several osteogenic differentiation related marker genes (RUNX2, COL-1A1, ALP, OCN, OPN, BSP, SOX9 and PPAR-γ) at day 3. Total RNA was extracted using Trizol (Invitrogen) according to the manufacturer’s instructions. Total RNA was reverse-transcribed into cDNAs using an Omniscript kit (Qiagen). mRNA expression was measured by using DyNamo SYBR1 Green qPCR kit (Takara). Primer sequences were designed as follows: RUNX2, forward primer 5′-GCGTCAACACCATCATTCTG-3′ and reverse primer 5′-CAGACCAGCAGCACTCCATC-3′; COL-1A1, forward primer 5′-GAGAGCATGACCGAT GGAT-3′ and reverse primer 5′- ATGTTTTGGTGGTTCAGGAGG-3′; ALP, forward primer 5′-GAGAGC ATGACCGATGGAT-3′ and reverse primer 5′-ATGTTTTGGTGGTTCAGGAGG-3′; OCN, forward primer 5′-AGAGCCCCAGTCCCCTACCC-3′ and reverse primer 5′-AGGCCTCCTGAAAGCCGATG-3′; OPN, forward primer 5′-CCGTTGCCCAGGACCTGAA-3′ and reverse primer 5′-TGTGGCTGTGGGTTTCAGCA-3′; BSP, forward primer 5′- TGCCTTGAGCCTGCCTGCTTCC-3′ and reverse primer 5′- CAAAATTAAAGCAGTCTTCATTTTG-3′; Y1 receptor, forward primer CTCGCTGGTTCTCATCGCTGTGGAACGG and reverse primer GCGAATGTATATCTTGAAGTAG; SOX9, forward primer 5′- TGGGCAAGCTCTGGAGACTTC-3′ and reverse primer 5′- ATCCGGGTGGTCCTTCTTGTG-3′; PPAR-γ, forward primer 5′- GCCGAGAAGGAGAAGC-3′ and reverse primer 5′-TGGTCAGCGGGAAGG-3′; PKA, forward primer 5′-AAAGAATGGGCAACCAGTG′ and reverse primer 5′-GCTGACCCCTAAAATAATG-3′; GAPDH, forward primer 5′-ACCACAGTCCATGCCATCAC-3′ and reverse primer 5′-TCCAC- CACCCTGTTGCTGTA-3′. Gene expression was calculated by normalizing its level to that of glyceraldehyde-3-phosphate dehydrogenase (GAPDH, an internal control) using the 2^−ΔΔCT^ methods.

### Western Blot

Total protein was extracted with a passive lysis kit (Promega) and quantified. Protein resolved by SDS-PAGE (Sigma) was then transferred to PVDF membranes. Membranes were incubated with blocking buffer (5% bovine serum albumin (BSA, Sigma)) for 2 h, and then incubated at 4°C overnight with antibodies against Y1 receptor (Abcam, No. 35336), RUNX2 (Millipore, No. 05-1478), p-PKA (Cell Signaling, No.9621), total PKA (Cell Signaling, No.4782), p-CREB (Cell Signaling, No.9198), total CREB (Cell Signaling, No.9193), p-caspase3 (Cell Signaling, No.9664), p-JNK (Santa Cruz, No. sc-6254), p-ERK (Cell Signaling, No.9101), p-P38 (Cell Signaling, No. 9211), Lamin B (Santa Cruz, No. sc-374015), LDH (Santa Cruz, No. sc-133123). These antibodies were diluted in TBST (50 mM Tris-HCl, pH 7.5, 150 mM NaCl, 0.05% (v/v) Tween 20). After washing, protein expression was detected by using a Western Chemiluminescent HRP Substrate Kit (Millipore), with GAPDH (Cell Signaling, No.8884) as the endogenous control. Quantitative densitometric values of the detected bands were quantified using the NIH Image J Software. We performed all Western blot assays at least three times.

### Nuclear and Cytosolic Fractionation

Nuclear and cytoplasmic fractionation was conducted by using the Nuclear and Cytoplasmic Extraction Reagents kit (Thermo Fisher Scientific), according to the manufacturer’s instructions. Briefly, cells collected by trypsinization were washed with ice-cold PBS and lysed with ice-cold 0.1% NP-40 (Sigma). The lysates were collected as the “whole cell lysate.” After centrifugation, the supernatants were collected as the “cytosolic fraction.” The pelleted “nuclear fraction” was further lysed in RIPA solution (Beyotime) with protease inhibitor cocktail tablets (Roche) and then collected. Protein in these fractions was analyzed by Western blot analysis.

### Small Hairpin RNA (shRNA) Plasmid Transduction

Methods for shRNA plasmid transduction and infection were previously described (8). In brief, hBMSCs were transfected with Y1 receptor shRNA plasmid (36098-SH, Santa Cruz), PKA shRNA plasmid (sc-39162-SH, Santa Cruz) and shCon by using Lipofectamine 2000 (Invitrogen) according to the manufacturer’s instructions. 48 h post-transfection, the cells were cultured in selective media including 4 μg/mL puromycin (Sigma). After the media being replaced every 3 days, the third passage of stable clones was collected in the following experiments. Resistant clones were pooled to generate polyclonal cells, and silence of targeted genes was verified by real-time PCR and Western blot analysis.

### Luciferase Reporter Assay

To assess luciferase activity, hBMSCs were transfected with pCRE-luciferase reporter plasmid (Agilent Technologies) and the internal control pRL-SV40 (Promega) using Lipofectamine 2000 (Invitrogen) according to the manufacturer’s instructions. Cells were cultured in osteogenic media for 3 days with shRNA or H-89 treatment. The target luciferase activity was analyzed using the Dual-Luciferase1 Reporter Assay System (Promega) by normalizing to the Renilla luciferase activity.

### Intracellular Cyclic Adenosine Monophosphate (cAMP) and PDE Activity Assay

Intracellular cAMP was quantitated using a direct cAMP ELISA kit (Enzo Life Sciences) according to the manufacturer’s instructions. Briefly, cells seeded into plates were scraped and lysed with 0.1 M HCl. Intracellular cAMP levels in the cell lysates were colorimetrically detected at 405 nm using the cAMP ELISA Kit. The final cAMP concentrations were calculated after being normalized by total protein content. For PDE activity assay, cell lysates were centrifuged and the supernatants were collected for further analysis. After purification by gel filtration, the supernatants were detected at 620 nm using a PDE Activity Assay Kit (Abcam) according to the manufacturer’s instructions. IBMX (a potent PDE inhibitor, 10 μM) was applied as the negative control. Quantification of the PDE activity was conducted with drawing a standard curve.

### Statistical Analysis

Experiments in this study were repeated in triplicate in each of the 5 donors (N=5), and the data were presented as mean ± standard deviation (SD). Statistical analyses were determined by one-way ANOVA followed by a *post hoc* Dunnett’s test or Student’s t-test. The SPSS 18.0 software was used for data management. Values of P < 0.05 were considered statistically significant.

## Results

### Effects of Y1 Receptor Regulation on the Viability and Differentiation of BMSCs

**Figure 1:**
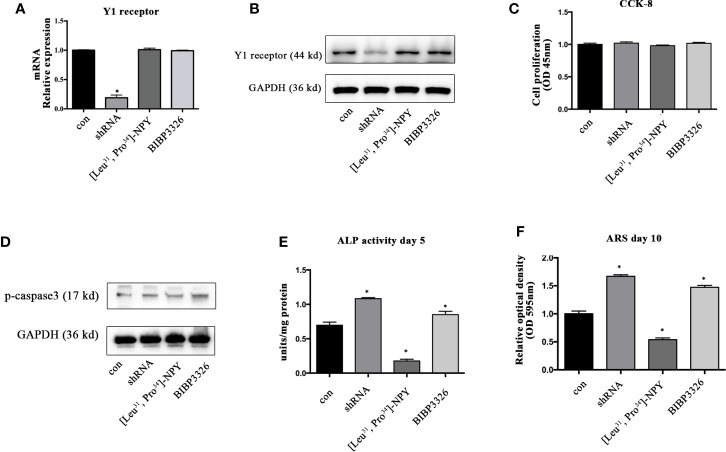
The effects of Y1 receptor signaling regulation on the viability and osteogenic differentiation of BMSCs. **(A, B)** BMSCs were treated with Y1 receptor shRNA, Y1 receptor agonist [Leu^33^, Pro^34^]-NPY and antagonist BIBP3326. Levels of Y1 receptor mRNA were detected by real-time PCR at day 3, with GAPDH as the internal control; protein expression of Y1 receptor were assessed by Western blot at day 3. **(C)** Cell proliferation was measured using a CCK-8 kit at day 3. **(D)** Cell apoptosis was measured by expression levels of phosphorylated casepase-3 (p-casepase3) by Western blot at day 3. **(E)** BMSCs were cultured in osteogenic media for 5 days. ALP activity was colorimetrically measured at 405 nm to determine the extent of osteoblast differentiation. **(F)** Alizarin Red S staining (ARS) was performed in BMSCs cultured in the osteogenic medium for 10 days. Quantification of Alizarin Red S staining was colorimetrically detected at 595 nm to determine the calcium nodule formation (N = 5). Data are presented as mean ± SD; *p < 0.05 compared to control. con: cells cultured in a normal or osteogenic medium; GAPDH, glyceraldehyde 3-phosphate dehydrogenase; OD, optical density (data presented in **A, C, E,** and **F** is pooled; data presented in **B, D** is pooled).

To regulate the expression levels of Y1 receptor in BMSCs, we used shRNA interference, pharmacological agonist [Leu^31^, Pro^34^]-NPY (10^−7^ M) and antagonist BIBP3226 (10^−7^ M). The level of Y1 receptor mRNA was significantly decreased after shRNA treatment, suggesting a high efficiency of shRNA interference. However, neither agonist nor antagonist of Y1 receptor influenced the mRNA expression of Y1 receptor (**Figure 1A**). The results of Western blot showed the similar trends in protein levels (**Figure 1B**). We then explored the effects of Y1 receptor regulation on cell proliferation and apoptosis. The results of CCK-8 demonstrated that gene and pharmacological regulation of Y1 receptor did not affect cell proliferation (**Figure 1C**). Since caspase-3 has been widely known as the key factor regulating cell apoptosis (25), we assessed cell apoptosis by detecting the phosphorylation of caspase-3 and found Y1 receptor did not affect cell apoptosis (**Figure 1D**). We then investigated the effects of Y1 receptor regulation on osteogenic differentiation in BMSCs by two measurements: ALP activity and calcium nodule formation. Inhibition of Y1 receptor signaling by shRNA or antagonist promoted osteogenic differentiation in BMSCs, as shown by an increase of alkaline phosphatase (ALP) activity and calcium nodules formation. In contrast, treatment with Y1 receptor agonist inhibited osteoblast differentiation of BMSCs with a low amount of ALP and calcium nodules (**Figures 1E, F**). These results suggested that inhibition of Y1 receptor signaling enhances osteoblast differentiation of BMSCs, which was consistent with the findings as we reported in the osteoblastic MC3T3 cells (8). Particularly, we found the pro-osteogenic effects of Y1 receptor deficiency by shRNA was relatively significant than that by pharmaceutical treatment, thus we used shRNA transfection to silenced Y1 receptor in the following study.

### Inhibition of Y1 Receptor Signaling Promoted RUNX2 Expression Through p-PKA Activation During Osteogenic Differentiation

**Figure 2:**
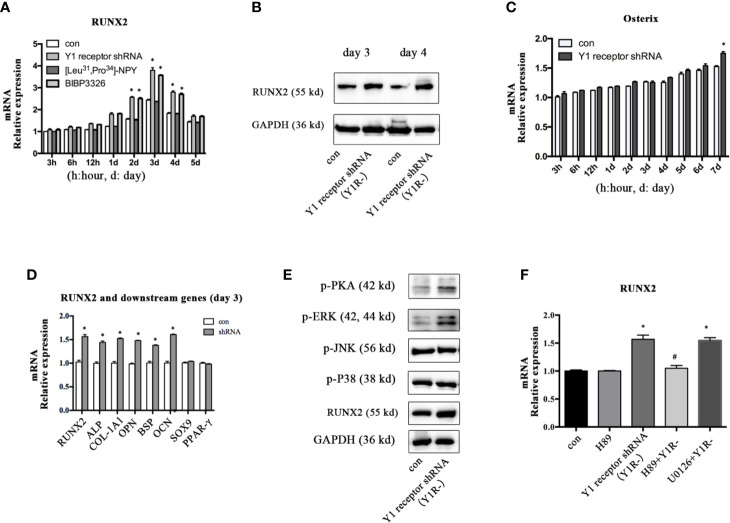
Inhibition of Y1 receptor signaling promoted RUNX2 expression and p-PKA during osteogenic differentiation. **(A)** Expression of RUNX2 mRNA in BMSCs during osteogenic differentiation were measured by real-time PCR. **(B)** BMSCs were incubated in osteogenic media for 3 and 4 days, and RUNX2 protein expression were measured by Western blot. **(C)** Expression of Osterix mRNA in BMSCs during osteogenic differentiation were measured by real-time PCR. **(D)** The levels of osteogenic genes were assessed by real-time PCR at day 3 during differentiation. **(E)** The levels of p-PKA, p-ERK, p-JNK, p-P38, and RUNX2 were measured by Western blot at day 3. **(F)** Expression of RUNX2 mRNA in BMSCs treated with Y1 deficiency, H-89 (a PKA inhibitor) and U0126 (a ERK inhibitor) were measured by real-time PCR at day 3. Real-time PCR was conducted using GAPDH as the internal control (N = 5). con: cells cultured in osteogenic media. Data are presented as mean ± SD; *p < 0.05 compared to control. GAPDH: glyceraldehyde 3-phosphate dehydrogenase (data presented in **A, B, D,** and **F** are pooled; data presented in **C, E** are representative). ^#^p < 0.05 compared to the group Y1 receptor shRNA.

To explore the molecular mechanisms mediating the osteogenic effects of Y1 receptor deficiency, we analyzed the expression levels of the transcriptional factor RUNX2 and Osterix, which were known to play critical roles during osteogenic differentiation. The expression levels of RUNX2 mRNA peaked on day 3 and gradually decreased thereafter during BMSCs osteogenesis. Inhibition of Y1 receptor signaling enhanced the levels of RUNX2 mRNA during BMSCs osteogenesis (**Figure 2A**). Consistently, RUNX2 protein expressions on day 3 and day 4 were increased by Y1 receptor deficiency (**Figure 2B**). The expression levels of Osterix gradually increased during differentiation, and there was a significant difference in the expression levels of Osterix with Y1 receptor shRNA treatment for 7 days (**Figure 2C**). Since RUNX2 acted as the upstream of Osterix (12), and the most significant changes in RUNX2 expression occurred on day 3 during differentiation, we thus explored the levels of target genes of RUNX2 on day 3. The target osteogenic genes of RUNX2, such as ALP, COL-1A1, OPN, BSP, and OCN, were also increased by Y1 receptor deficiency on day 3 (**Figure 2D**). It has been reported that RUNX2 activity was modulated by several upstream signalings including PKA (26, 27), MAPK components (ERK, P38 and JNK pathways) (27, 28). We further investigated how Y1 receptor deficiency promoted RUNX2 expression by detecting these signaling pathways. The levels of p-P38 and p-JNK signaling proteins were not affected by Y1 receptor knockdown. While the levels of p-PKA and p-ERK signaling proteins were increased by Y1 receptor knockdown (**Figure 2E**), thus PKA and ERK signaling pathways were activated by Y1 receptor deficiency. Moreover, H-89 (a PKA inhibitor, 10^−5^ M) but not U0126 (a ERK inhibitor, 10^−5^ M) reversed RUNX2 induction by Y1 receptor deficiency (**Figure 2F**). These results demonstrated Y1 receptor deficiency enhanced RUNX2 expression through PKA pathway, which might be the main pathway mediating the osteogenic effects of Y1 receptor deficiency.

**Figure 3:**
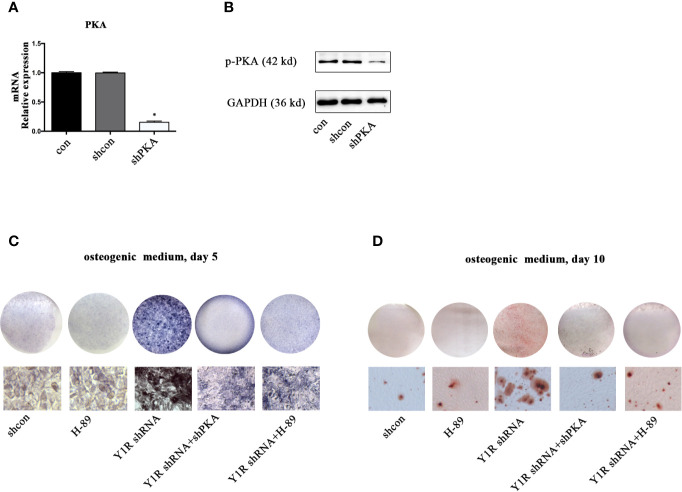
PKA inhibition abolished osteogenic effects of Y1 receptor deficiency in BMSCs. BMSCs were cultured in osteogenic media, with or without Y1 shRNA, shPKA and H-89 (0.1μM). ALP staining was performed on day 5 and Alizarin Red S staining was performed on day 10. **(A, B)** PKA expressions in mRNA and protein levels were assessed by real-time PCR and Western blot at day 3. **(C)** BMSCs treated with shcon, Y1 receptor shRNA, shPKA, and H-89 were cultured in osteogenic media; ALP staining was performed on day 5. **(D)** BMSCs treated with shcon, Y1 receptor shRNA, shPKA, and H-89 were cultured in osteogenic media; ARS staining was performed on day 10. Real-time PCR was conducted using GAPDH as the internal control (N = 5). con: cells cultured in osteogenic media (data presented in a is pooled; data presented in **B–D** are representative). *p < 0.05 compared to control.

We then silenced PKA by using shRNA to verify this hypothesis. The results of real-time PCR and Western blot showed a high efficiency of shPKA (**Figures 3A, B**). Both genetic and pharmacological blockade of PKA signaling reduced ALP activity and mineralization of BMSCs (**Figures 3C, D**), indicating that Y1 receptor deficiency triggers osteogenic differentiation through activation of the PKA pathway.

### PKA Inhibitor Abolished Phosphorylation of PKA Downstream Signaling Induced by Y1 Receptor Deficiency

**Figure 4:**
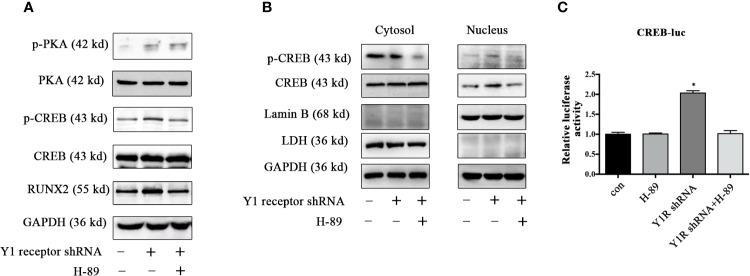
PKA inhibitor abolished phosphorylation of PKA downstream signaling induced by Y1 receptor deficiency. BMSCs were incubated in osteogenic media for 5 days. **(A)** Expression levels of PKA, CREB, and RUNX2 were quantified by Western blot analysis. **(B)** Cell lysates were collected and fractionated into nuclear and cytosolic extracts. Expression levels of p-CREB and CREB were analyzed by Western blot analysis. LDH was used as a control for cytosol protein samples, and Lamin B was used as a control for nuclear protein samples. **(C)** CREB transcriptional activity was assessed by luciferase reporter assay in BMSCs treated with Y1 shRNA or H-89 for 5 days during osteoblast differentiation. The luciferase activity of the cell lysates was measured using Dual-luciferase reporter assay system with normalization to Renilla luminescence (N = 5). Data are presented as mean ± SD; *p < 0.05 compared to control. GAPDH: glyceraldehyde 3-phosphate dehydrogenase. con: cells cultured in osteogenic media. CREB-luc: cAMP-response element binding protein (CREB) luciferase reporter plasmid (data presented in **A, B** are representative; data presented in **C** are pooled).

Since CREB was the main downstream signaling of PKA, we assessed the expression of p-CREB and found Y1 receptor knockdown upregulated the levels of p-PKA and p-CREB in BMSCs during osteogenic differentiation for 5 days (**Figure 4A**). Moreover, H-89 (a potent PKA inhibitor) suppressed RUNX2 and p-CREB expression induced by Y1 receptor deficiency (**Figure 4A**), indicating that PKA signaling mediated RUNX2 and p-CREB expression induced by Y1 receptor deficiency. We then assessed the cellular location of p-CREB, which was associated with nuclear import (29). The results showed that the p-CREB nuclear translocation was activated by Y1 receptor knockdown and was reversed by PKA inhibition during BMSCs differentiation (**Figure 4B**). To further investigate whether CREB transcription levels were regulated by CREB nuclear translocation induced by Y1 receptor deficiency, we conducted luciferase reporter assay thereafter. The results demonstrated that Y1 receptor deficiency increased transcription activity of CREB, while PKA inhibitor blocked this effect (**Figure 4C**). Altogether, our data indicated that PKA was the signaling pathway through which Y1 deficiency enhanced osteogenic differentiation.

### Upregulation of p-PKA Depended on the Induction of Intracellular cAMP

**Figure 5:**
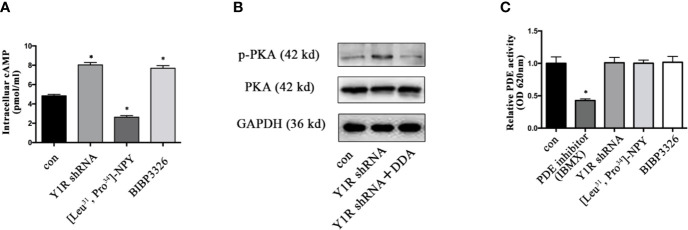
Y1 receptor deficiency increased the level of intracellular cAMP. **(A)** BMSCs were treated with Y1 receptor shRNA, [Leu^31^,Pro^34^]-NPY and BIBP3226 for 15 min. Intracellular cAMP production was measured by ELISA. **(B)**: The expression levels of p-PKA were detected in BMSCs treated with Y1 receptor shRNA and DDA (a cAMP inhibitor, 10^−5^ M) by Western blot. **(C)** PDE activity was measured in BMSCs treated with IBMX (isobutylmethylxanthine, a PDE inhibitor, 10 µM), Y1 receptor shRNA, [Leu^31^, Pro^34^]-NPY and BIBP3226. N=5; Data are presented as mean ± SD; *p < 0.05 compared to control. con: cells cultured in a normal medium. PDE: phosphodiesterases, OD: optical density (data presented in **A, C** are pooled; data presented in **B** are representative).

It was well established that cAMP induction enhanced gene expression of bone specific growth factors (15, 30), and PKA phosphorylation depended on cellular cAMP (31), we thus determined whether PKA signaling activation induced by Y1 deficiency was through cellular cAMP during BMSCs differentiation. The intracellular cAMP levels were increased in BMSCs with Y1 receptor knockdown (**Figure 5A**). Blockade of intracellular cAMP by adenylyl cyclase (AC) inhibitor 2′, 3′-dideoxyadenosine (DDA, 10^−5^ M, Sigma) (32, 33) significantly abolished p-PKA activation by Y1 receptor deficiency (**Figure 5B**). It has been reported phosphodiesterases (PDEs) was a hydrolase targeting cAMP (34); we thus evaluated its expression levels in BMSCs differentiation. The results showed no significant change of PDEs expression in BMSCs treated with Y1 receptor knockdown (**Figure 5C**). In conclusion, activation of the p-PKA by Y1 receptor deficiency was dependent on intracellular cAMP induction, without dependency on PDEs regulation.

## Discussion

By using genetic and pharmacological approaches, our results reveal that Y1 receptor inhibition enhances osteogenesis of BMSCs through PKA signaling. This effect is mediated by nucleus translocation of p-CREB, and thus promotes RUNX2 expression. Particularly, Y1 receptor inhibition likewise increases the levels of intracellular cAMP, suggesting that intracellular cAMP mediates the activation of PKA signaling. In conclusion, we propose that inhibition of Y1 receptor promotes osteogenesis in BMSCs *via* cAMP/PKA/CREB pathway.

Neuropeptide Y (NPY) and its receptors are not only neuronal regulators of energy homeostasis (35), but are also known to be involved in bone homeostasis (36). As the only Y receptor expressed by BMSCs and mature osteoblast in situ (7), Y1 receptor has a negative effect on osteoblastic differentiation and bone formation. Germ-line Y1 receptor deletion mice results in a high bone mass phenotype with increased mineral node apposition (5). Furthermore, osteoblast specific knockout of Y1 receptor also enhances the differentiation potential of BMSCs and results in increased bone formation in mice, suggesting a critical role of Y1 receptor in osteoblast (7). Practically, RUNX2, a key transcription factor for osteogenesis, is upregulated by Y1 receptor silence in both the bones tissues (7) and MC3T3−E1 osteoblast cells (37). RUNX2 is critical for early-stage differentiation of BMSCs, which drives the expression of many osteoblast-specific genes (38, 39). RUNX2 knockout in mice results in lack of mature osteoblasts and decreased bone formation (11). Thus RUNX2 is one of the most important regulators in osteogenic differentiation, and we detected its activity in hBMSCs during differentiation. This study shows that RUNX2 expression and osteoblastic differentiation of hBMSCs are enhanced by Y1 receptor shRNA, which is consistent with previous reports.

To further determine the signaling pathways through which Y1 deficiency enhances osteogenic differentiation, we silence Y1 receptor by shRNA in BMSCs. The proliferation and apoptosis of BMSCs were not affected by Y1 receptor regulation, implying that Y1 receptor did not impact the viability of BMSCs. Thereafter, the osteogenic effect of Y1 receptor on BMSCs was independent of enhanced self-renewal capacity. Given the critical role of RUNX2 and Osterix in osteogenic differentiation, we then scanned their expression levels in BMSCs. Y1 receptor deficiency significantly increased RUNX2 expression at the early stage of osteogenic differentiation, which was consistent with previous reports (7, 37). In contrast, Y1 receptor deficiency enhanced Osterix expression at the relative late stage of osteogenic differentiation. Since RUNX2 is thought to be the upstream of Osterix (12), this study focuses on the expression level of RUNX2. We then hypothesize that the osteogenic effect of Y1 receptor deficiency is through upregulation of RUNX2 in BMSCs, and further explore the underlying mechanisms.

Y1 receptor, interacts with G_i/o_ G protein, has been reported to play an inhibitory role in adenylate cyclase (AC) activity and cAMP production (1, 19), while cAMP promotes osteoblast differentiation *via* PKA and CREB activation (15, 40). Moreover, cAMP production induced by Y1 receptor deficiency activates protein kinases A (PKA) and CREB phosphorylation in islets β-cells (21). It has been shown that RUNX2 activity was regulated by cAMP/PKA pathways and MAPK components (ERK, P38 and JNK pathways) (26–28). We next detected the expression levels of cAMP/PKA pathways as well as p38, JNK, and the ERK signaling in BMSCs treated with Y1 receptor silence during osteogenic differentiation. The result showed the upregulated expression of RUNX2 induced by Y1 receptor deficiency was through cAMP/PKA pathways, independent of MAPK components.

In this study, we find that cAMP production is increased by Y1 receptor deficiency in BMSCs. Since the expression of intracellular cAMP is regulated by adenylate cyclase (AC) and phosphodiesterases (PDE) activity (41), we detected the PDE activity and found it was not affected by Y1 receptor regulation. Therefore, the increased intracellular cAMP levels are rather dependent on regulation of AC than PDE activity. Y1 receptor deficiency enhances osteogenic differentiation *via* direct effects on cAMP production. Further support for this concept comes from studies where we could show that protein levels of p-CREB are significantly upregulated in Y1 receptor silence BMSCs.

Taken together, these data demonstrate that reduced Y1 receptor signaling exerts osteogenic effects in BMSCs by activation of RUNX2 *via* the cAMP/PKA/CREB pathway. Regulation of Y1 receptor signaling might offer promise as a potential therapy for osteoporosis and other bone loss conditions.

There are some limitations in this study. Primary human cells used in this study are known to produce high standard deviations. Moreover, this study focuses on the expression of RUNX2 in the early stage of differentiation, and hypothesizes that changes in RUNX2 as the cause of osteogenesis regulation by Y1 receptor. Since osteoblast differentiation takes place over a long timeframe, and transcription factor such as Osterix may also play an important role in the later stage of differentiation. This will be an issue we need to further study.

## Data Availability Statement

The raw data supporting the conclusions of this article will be made available by the authors, without undue reservation.

## Ethics Statement

The studies involving human participants were reviewed and approved by Institutional Review Board (IRB) of the Second Affiliated Hospital, Wenzhou Medical University (IRB No. 2018-14). The patients/participants provided their written informed consent to participate in this study.

## Author Contributions

WY, S-DJ, and X-YP conceived and designed the experiments. WY, W-NX, and F-CC performed the experiments. S-LD and P-BC acquired and analyzed the data. WY drafted the manuscript. LY and X-YP helped perform the analysis with constructive discussions and revised the manuscript. All authors contributed to the article and approved the submitted version.

## Funding

This work was supported by National Natural Science Foundation of China (Grant No.: 81672206); Zhejiang Province Technology Project (grant number 2015C33209); and Wenzhou Technology Project (grant number Y20150243).

## Conflict of Interest

The authors declare that the research was conducted in the absence of any commercial or financial relationships that could be construed as a potential conflict of interest.
